# Effects of Acute Gamma Irradiation on Physiological Traits and Flavonoid Accumulation of *Centella asiatica*

**DOI:** 10.3390/molecules16064994

**Published:** 2011-06-17

**Authors:** Sina Siavash Moghaddam, Hawa Jaafar, Rusli Ibrahim, Asmah Rahmat, Maheran Abdul Aziz, Elizabeth Philip

**Affiliations:** 1 University Putra Malaysia, 43400 UPM Serdang, Selangor, Malaysia; Email: sina.siavash@simedarby.com (S.S.M.); asmah@medic.upm.edu.my (A.R.); maheran@agri.upm.edu.my (M.A.A.); 2 Agrotechnology & Biosciences Division, Malaysian Nuclear Agency, Bangi 43000 Kajang, Selangor, Malaysia; Email: rusli_ibrahim@nuclearmalaysia.gov.my; 3 Forestry and Environment Division, Forest Research Institute Malaysia (FRIM), Kepong, 52109 Kuala Lumpur, Malaysia; Email: philip@frim.gov.my

**Keywords:** *Centella asiatica*, gamma irradiation, total flavonoid, photosynthesis

## Abstract

In the present study, two accessions of *Centella asiatica* (CA03 and CA23) were subjected to gamma radiation to examine the response of these accessions in terms of survival rate, flavonoid contents, leaf gas exchange and leaf mass. Radiation Sensitivity Tests revealed that based on the survival rate, the LD_50_ (gamma doses that killed 50% of the plantlets) of the plantlets were achieved at 60 Gy for CA03 and 40 Gy for CA23. The nodal segments were irradiated with gamma rays at does of 30 and 40 Gy for *Centella asiatica* accession ‘CA03’ and 20 and 30 Gy for accession ‘CA23. The nodal segment response to the radiation was evaluated by recording the flavonoid content, leaf gas exchange and leaf biomass. The experiment was designed as RCBD with five replications. Results demonstrated that the irradiated plantlets exhibited greater total flavonoid contents (in eight weeks) significantly than the control where the control also exhibited the highest total flavonoid contents in the sixth week of growth; 2.64 ± 0.02 mg/g DW in CA03 and 8.94 ± 0.04 mg/g DW in CA23. The total flavonoid content was found to be highest after eight weeks of growth, and this, accordingly, stands as the best time for leaf harvest. Biochemical differentiation based on total flavonoid content revealed that irradiated plantlets in CA23 at 20 and 30 Gy after eight weeks contained the highest total flavonoid concentrations (16.827 ± 0.02; 16.837 ± 0.008 mg/g DW, respectively) whereas in CA03 exposed to 30 and 40 Gy was found to have the lowest total flavonid content (5.83 ± 0.11; 5.75 ± 0.03 mg/g DW). Based on the results gathered in this study, significant differences were found between irradiated accessions and control ones in relation to the leaf gas. The highest PN and gs were detected in CA23 as control followed by CA23 irradiated to 20Gy (CA23G20) and CA23G30 and the lowest PN and gs were observed in CA03 irradiated to 40Gy (CA03G40). Moreover, there were no significant differences in terms of PN and gs among the irradiated plants in each accession. The WUE of both irradiated accessions of *Centella asiatica* were reduced as compared with the control plants (p < 0.01) while Ci and E were enhanced. There were no significant differences in the gas exchange parameters among radiated plants in each accession. Moreover, malondialdehyde (MDA) of accessions after gamma treatments were significantly higher than the control, however, flavonoids which were higher concentration in irradiated plants can scavenge surplus free radicals. Therefore, the findings of this study have proven an efficient method of in vitro mutagenesis through gamma radiation based on the pharmaceutical demand to create economically superior mutants of *C. asiatica*. In other words, the results of this study suggest that gamma irradiation on *C. asiatica* can produce mutants of agricultural and economical importance.

## 1. Introduction

It is important to note that the daily intake of flavonoids in normal food, particularly fruits and vegetables, is 1–2 g. Modern physicians are increasing their use of pure flavonoids to treat many important common diseases due to their proven ability to restrain specific enzymes, to stimulate a number of hormones and neurotransmitters, and to scavenge free radicals [1]. *Centella asiatica* (L.) Urban (Family: Apiaceae) is a significant traditional medicinal plant. Consumption of *Centella asiatica* as a nutraceutical has been shown to be a significant way to achieve the desired quantity of flavonoids from plants, however the flavonoids concentration in *Centella asiatica* is still relatively low.

The amount of secondary metabolites in plants can be boosted by elicitation. Elicitors can be grouped into two categories: (i) abiotic elicitors (e.g., irradiation, methyl jasmonate, salts of heavy metals and various chemicals), and (ii) biotic elicitors (e.g., microbe-derived molecules that stimulate secondary metabolism such as fungal cell wall, glycoproteins, and polysaccharides) [2].

Several energy rays are widely used in mutation breeding, such as X-, β-, and γ-rays, and neutrons, and protons. Gamma rays were reported to be the most efficient ionizing radiation of creating mutants in plants as they can induce high mutation numbers in plants. They could also modify physiological characteristics to create new mutants with improved properties that can produce higher amounts of commercially essential metabolites, developing varieties that are agriculturally and economically significant, and contain high productivity potential [3,4,5]. 

Gamma radiation can interacts with atoms and molecules to create free radicals in cells that are able to modify important components of plant cells. These radicals have been demonstrated to affect the morphology, anatomy, biochemistry, and physiology of plants, depending on the irradiation dosage. The effects consist of changes in the plant cellular structure and metabolism, e.g., dilation of thylakoid membranes, change in photosynthesis, modulation of the antioxidative system, changes in malondialdehyde (MDA) levels as marker of free radicals and enhancement of phenolic compounds [6,7,8]. 

It is important to highlight that limited information exists on the potential and extent of enhancing production of secondary metabolites using physical elicitors such as gamma radiation, even though many of the biological effects of gamma radiation have received considerable attention [9]. Moreover, few studies have documented the effects of ionizing radiation on photosynthesis. Therefore, it is important to examine effects of ionizing radiation on the photosynthetic system. In light of this, this study was carried out to stimulate flavonoids production in the leaves and whole plant of *Centella asiatica* using gamma irradiation as physical elicitor. 

## 2. Results and Discussion

### 2.1. The Radiation Sensitivity Test

**Figure 1:**
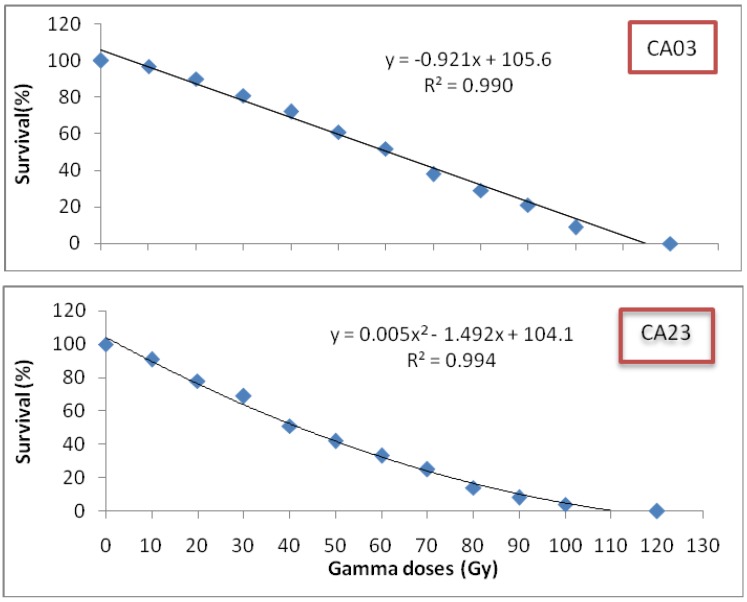
Radiation effect on survival rate (%) for *C. asiatica.*

The sensitivity of *Centella asiatica* to radiation was evaluated by comparing the survival rate (%) between irradiated and non-irradiated plantlets. Highly significant differences (P ≤ 0.01) were observed among the different doses of irradiation. The plantlet survival rate kept decreasing with increasing irradiation dosage for three weeks after irradiation, as illustrated in Figure 1. 

The accession CA03 was found to be less sensitive to gamma irradiation than the CA23 one. At 50% (LD_50_) survival rate (%), the gamma doses administered were 60 Gy for CA03 and 40 Gy for CA23. The plantlets died in the seventh week after treatment. It should be noted that a reduction in the survival rates of *Centella asiatica* was observed with increasing doses of gamma irradiation.According to Salvana [10], *Polianthes tuberosa* appeared to be less sensitive to high levels of gamma irradiation. This was confirmed by the finding that when higher doses were applied, even at the 100 Gy level, a 10% survival rate was observed and the plantlets died in the fifteenth week. Additionally, when seeds of red pepper were first gamma-irradiated, the resultant plant growth was stimulated at 2 to 8 Gy but was scarcely affected at 16 Gy [7]. In contrast, Ling *et al.* [11] found that plant growth was stimulated at 10 Gy and that inhibition occurred at radiation levels above 10 Gy. There are no large variations in growth stimulation and inhibition between these two studies. This implies that irradiation enhances plant sensitivity to gamma irradiation. This could be caused by the reductions in the amounts of endogenous growth regulators, particularly the cytokines, as a consequence of break down, or lack of synthesis due to irradiation [11].

### 2.2. Determination of Plant Biomass and Total Flavonoid Content

**Figure 2:**
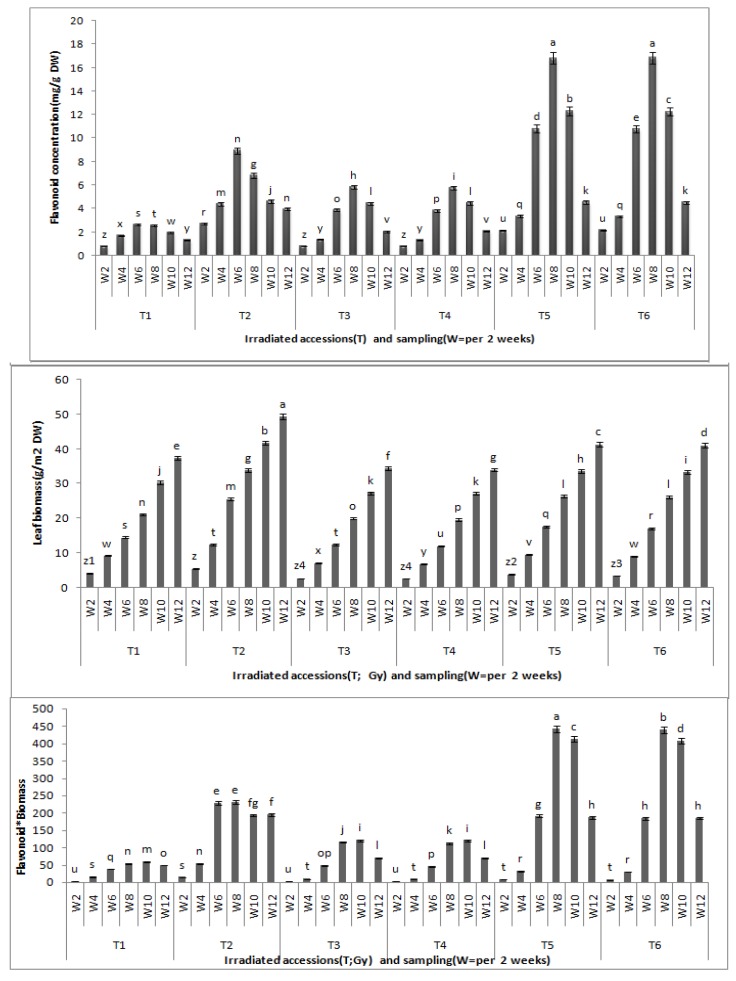
Effects of gamma irradiation on leaf total flavonoid, leaf biomass and flavonoid * leaf biomass in *C.asiatica*. n = 4 (T1 = CA03 (Control); T2 = CA23 (Control); T3 = CA03 irradiated to 30 Gy; T4 = CA03 irradiated to 40 Gy; T5 = CA23 irradiated to 20 Gy; T6 = CA23 irradiated to 30 Gy; W2 = 2 weeks after planting; W4 = 4 weeks after planting; W6 = 6 weeks after planting; W8 = 8 weeks after planting; W10 = 10 weeks after planting; W12 = 12 weeks after planting).

Statistical tests of flavonoid contents of *Centella asiatica* leaves between the growth stages and between the gamma radiation doses revealed significant differences at (P < 0.05). The total flavonoid content was found to be highest after eight weeks of growth, and this, accordingly, stands as the best time for leaf harvest. Biochemical differentiation based on total flavonoid content revealed that irradiated CA23 plantlets at 20 and 30 Gy after eight weeks contained the highest total flavonoid concentrations (16.827 ± 0.02; 16.837 ± 0.008 mg/g DW, respectively) whereas CA03 exposed to 30 and 40 Gy was found to have the lowest total flavonid content (5.83 ± 0.11; 5.75 ± 0.03 mg/g DW) (Figure 2).

Comparing total flavonoid contents of control with the irradiated plantlets, results demonstrate that the irradiated plantlets exhibited significantly greater total flavonoid contents (in eight weeks) than the control where the control also exhibited the highest total flavonoid contents in the sixth week of growth: 2.64 ± 0.02 mg/g DW in CA03 and 8.94 ± 0.04 mg/g DW in CA23, respectively. These contents were 54.7% and 46.8% lower, respectively, than the total flavonoids contents of the irradiated plants in their eighth week of growth. However, there were no significant differences in total flavonoid contents between the irradiated plants with 30 and 40 Gy in CA03 and between those irradiated with 20 and 30 Gy in CA23. From the results obtained in the present study, it can be concluded that plants irradiated with gamma radiation showed the higher total flavonoid content as compared to non-irradiated plants. Moreover, at the eighth week of growth, the irradiated plants were found to have the highest flavonoid contents. 

Flavonoid biosynthesis is stimulated by enhancing phenylalanine content and phenylalanine ammonia-lyase activity (PAL). Flavonoids are secondary metabolites broadly distributed in plants. They result from the addition of malonyl CoA to the phenylpropanoid molecule coumaroyl CoA [12]. These polyphenolic compounds are categorized by two aromatic rings (A and B rings) linked via a heterocycle (C ring). They are grouped based on the degree of oxidation of the C ring and comprise flavonols, anthocyanins, and flavan-3-ols. The carbon atoms on the B and C rings are from phenylalanine that is from the shikimic acid way. As primary factors in flavonoid synthetic pathway, the phenylalanine content influences flavonoid content directly.

Flavonoid biosynthesis, which is a significant step in the phenylpropanoid pathway, is the conversion of phenylalanine to cinnamic acid in the presence of PAL as catalyst. The activity of PAL affects the flavonoid synthesis in response to irradiation (gamma and UV-B stress) whereas the flavonoids alleviate the damage induced by the irradiation stress. For instance, the flavonoid content of soybean seedlings increases in response to UV-B radiation. With prolonged stress, the damage induced by UV-B radiation to soybean seedlings cannot be ameliorated completely by increased flavonoid concentrations. As a result, chlorophyll synthesis is inhibited and assimilation in leaves is constantly reduced by the UV-B stress, which ultimately results in a decline in the efficiency of the secondary metabolism biosynthesis system. Hence, biosynthesis and accumulation of flavonoids decrease [13].

A significant difference was observed between irradiated and non-irradiated plants for total biomass where the former had lower leaf biomass than the control. The data obtained after 12 weeks of growth showed that CA23 achieved the highest leaf dry weight (49.4 ± 0.2 g/m^2^ DW) and the CA03 at 40 Gy (CA03G40) had the slowest growth (34.0 ± 0.2 g/m^2^ DW). Furthermore, the flavonoid*biomass (flavonoids content multiplied by leaf biomass at the same harvest time) were significantly different between the irradiated accessions and the control. The CA23 had the highest flavonoid*biomass (442.56 ± 8.53) in the eighth week and at 20 Gy (CA23G20). On the other hand, no significant differences were observed in the flavonoid*biomass between the irradiated plants of each accession (Figure 2). 

It can be inferred that the biomass of *Centella asiatica* leaves was significantly inhibited by γ-radiation as compared with the control (p < 0.01). Nonetheless, there were no significant differences in leaf biomass between the irradiated plants in each of accession at the same harvest time.

Irradiation of Arabidopsis caused increases in the levels flavonoids, which accumulated in the aerial parts of the plants [14]. Jia and Li [15] found that plant height, number of first-class branches, and rhizome biomass in buckwheat were restrained drastically by gamma irradiation (p < 0.01). Reduction in growth of red stem buckwheat mutants, in contrast with increases in dry mass of soybean and secondary metabolite of *Lithospermum erythrorhizon*, may possibly be the reason for slow cell division; lower hormone synthesis; unusual nutrimental transportation; and metabolic disorders by apical meristem injure under γ-irradiation [15].

Banerji and Datta [16] and Shukla and Datta [17] reported a decline in Chrysanthemum height and numbers of branches and leaves when it was irradiated with low doses of gamma rays (1.5, 2, and 2.5 k-rad). Likewise, Ramachandran and Goud [18] showed that higher doses of gamma irradiation (G.I). (40-120 k-rad) decreased plant height, number of leaves and the branching capacity of safflower. A diminution in plant height, branches number, leaves number and size was observed when root cuttings of Chrysanthemum were irradiated with 20 or 25 k-rad gamma rays (16).

The growth of Arabidopsis seedlings exposed to low-dose gamma irradiation (1 or 2 Gy) was faintly increased compared with that of the control, while the seedling growth was perceptibly decreased by a high-dose irradiation of 50 Gy. Although no certain explanations for the stimulatory effects of low-dose gamma irradiation are accessible until now, a number of researchers support the assumption that low dose irradiation will stimulate growth by altering the hormonal signalling network in the plant cells or via enhancing the antioxidative capacity of the cells to simply overcome daily stress factors such as variations in light intensity and temperature in the growing medium/environment [7]. By contrast, the growth inhibition induced through the high-dose irradiation has been attributed to the cell cycle arrest at the G2/M phase during somatic cell division and/or to a variety of damages in the entire genome [19]. 

The association between growth of irradiated plants and dose of gamma irradiation has been demonstrated by investigating the morphological alteration and seedling growth of the irradiated plants. No significant morphological abnormalities were observed in the phenotype of the plants irradiated with relatively low doses (1–5 Gy) of gamma rays whereas a high-dose (50 Gy) irradiation restrained seedling growth considerably. As an illustration, Kim *et al.* [9] claimed that the growth of red pepper was raised at 2 and 4 Gy but slowed down at 8 and 16 Gy and that the level of stress resistance of gamma-irradiated plants may depend on the species/cultivar or stress circumstances [7].

### 2.3. Leaf Gas Exchange

**Figure 3:**
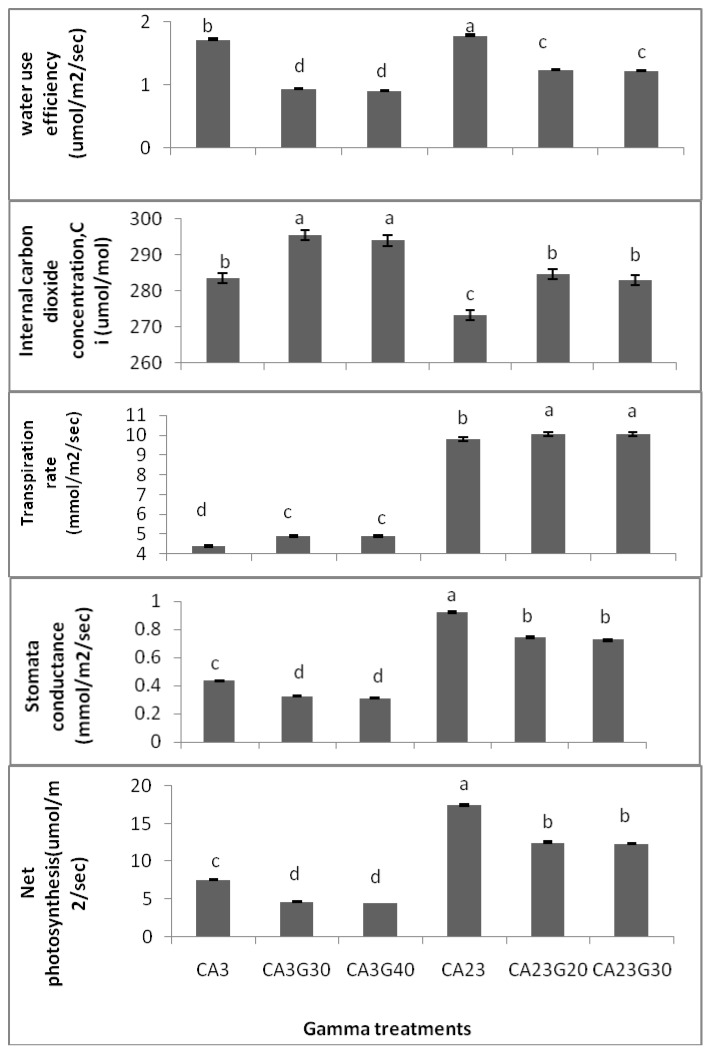
Effects of gamma irradiation on leaf gas exchange and WUE of irradiated and non irradiated accessions n = 4 (CA03G30 = CA03 irradiated to 30 Gy; CA03G40 = CA03 irradiated to 40 Gy; CA23G20 = CA23 irradiated to 20 Gy; CA23G30 = CA23 irradiated to 30 Gy).

In the eighth week of growth, net photosynthetic rate (PN), stomatal conductance (gs), intercellular CO_2_ concentration (C_i_), and transpiration rate (E) were determined by a portable infrared photosynthesis system LI-6400 (LI-COR, Lincoln, NE, USA) from 8:30 am to 10:30 am. Photosynthetic photon flux density (PPFD) and leaf temperature were maintained at 1000 μmol m^–2^ s^–1 ^and 30 °C, respectively. Water use efficiency (WUE) was calculated according to Penuelas *et al.* [20]. Based on the results gathered in this study, significant differences were found between irradiated accessions and control ones in relation to the leaf gas. As shown in Figure 3, the highest PN and gs were detected in CA23 as control (17.45 ± 0.03, 0.923 ± 0.002, respectively), followed by CA23 at 20 Gy (CA23G20) (12.48 ± 0.06, 0.742 ± 0.011) and CA23 at 30 Gy (CA23G30) (12.33 ± 0.06, 0.725 ± 0.009) and the lowest PN and gs were observed in CA03 at 40 Gy (CA03G40) (4.44 ± 0.04, 0.31 ± 0.003; respectively). Moreover, there were no significant differences in terms of PN and gs among the irradiated plants in each accession. The highest C_i_ (intercellular carbon dioxide) was found in CA03 (285.0 ± 0.8), while the lowest was found in CA23 (274.0 ± 0.8).

There were significant differences between irradiated accessions and control ones in terms of C_i_, E and WUE. The WUE of both irradiated accessions of *Centella asiatica* were reduced as compared with the control plants (p < 0.01) while Ci and E were enhanced. The transpiration rate increased but C_i_ and gs decreased in response to γ-radiation (Figure 3). There were no significant differences in the gas exchange parameters among radiated plants in each accession. However, the CA03G30 was observed to have the highest C_i_ (295.5 ± 0.6). Still, this concentration was not significantly different from that of CA03G40 (294.0 ± 0.3) while CA23 had the lowest C_i_ (273.25 ± 1.03). Meanwhile, CA23G30 was found to have the highest E (10.075 ± 0.005) while the lowest E was detected in CA03 (4.375 ± 0.015). As illustrated in Figure 3, the highest WUE was found in CA23 (1.783 ± 0.006) and the lowest was detected in CA03G40 (0.905 ± 0.006).

In buckwheat mutants’ reduction of Chl contents showed that Chl accumulation was reduced by γ-radiation, which stimulated activities of chlorophyllase, increased degradation of Chl, and ultimately reduced the photosynthetic activity of the plants. Although photosynthetic efficiency and biomass were declined notably, plants could acclimate to the varying growth environmental conditions through a defensive mechanism (secondary metabolites) against over-damage of the PSII reaction centres and excessive reduction of photosynthetic productivity. Jia and Li [15] showed that the photosynthetic rate of buckwheat fell down while that of *Capsicum annuum* accelerated by effect of a low dose of γ-radiation.

Whether stomatal or non-stomatal restriction is the significant factor in reducing PN could be judged upon by the altering patterns of both C_i_ and gs [21]. If both C_i_ and PN reduced through a lowering of gs, the decline of PN would be caused mostly via stomatal limitation. And it would be caused by non-stomatal limitation when C_i_ increased or remained almost unchanged with low PN and gs. In view of the above theory, the photoinhibition of buckwheat leaves was influenced by stomatal and non-stomatal limitation. On the one hand, stomata closure and gs decline due to rapid transpiration and excessive loss of water restricted the quantity of CO_2_ entering the plant leaves through lower C_i_ which consequently reduced photosynthesis rate due to a lack of photosynthetic substrate. On the other hand, the PN still declined through higher C_i_ values owing to lower gs as a consequence of limited carboxylation efficiency and CO_2_ assimilation because of the excessive accumulation of free radicals in mesophyll cells by irradiation [22]. Ursino *et al.* [23] revealed that the diminished rates of CO_2_ uptake in soybean plants were the result of a radiation impact on the photosynthetic apparatus rather than to increased stomatal resistance or to accelerated CO_2_ evolution from dark respiration. Furthermore, decreases in the rate of shoot growth and leaf expansion were apparent even at a dose of 750 rads.

### 2.4. Determination of Lipid Oxidation (Malondialdehyde (MDA))

**Figure 4:**
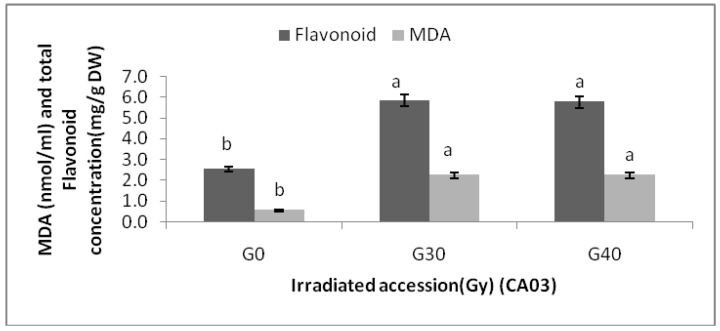
Flavonoid content and MDA in irradiated and non-irradiated accessions of CA03 n = 3 (CA03G30 = CA03 irradiated to 30 Gy; CA03G40 = CA03 irradiated to 40 Gy). Notice: Each observation compared with its counterparts.

**Figure 5:**
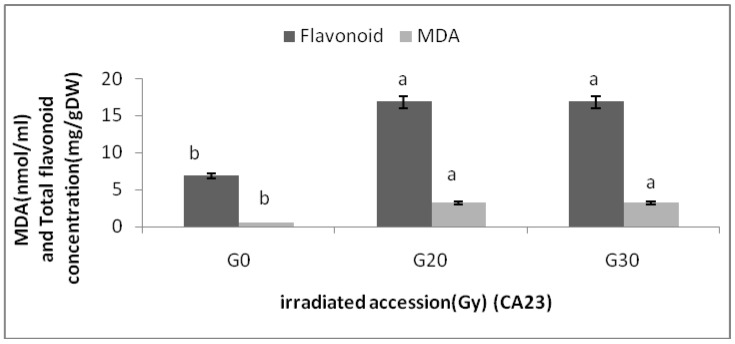
Flavonoid content and MDA in irradiated and non-irradiated accessions of CA23 n = 3 (CA23G20 = CA23 irradiated to 20 Gy; CA23G30 = CA23 irradiated to 30 Gy). Notice: Each observation compared with its counterparts (e.g, MDA compared with MDA in different accessions).

Changes in MDA content of the two *Centella asiatica* accessions under different gamma treatments were determined. The MDA contents of the *Centella asiatica* accessions after gamma treatments were significantly higher than the control. However, no significant differences in MDA contents among the irradiated plants in both accessions were detected. The highest MDA (3.20 ± 0.00 nmol/mL) was observed in CA23G30, while the lowest were detected in CA03 (0.543 ± 0.000 nmol/mL) and CA23 (0.540 ± 0.000 nmol/mL). For additional information about the relationships between MDA and flavonoids, graphs (Figure 4 and Figure 5) are provided, where it can be seen that a higher levels of MDA, flavonoid content was higher. 

Peng and Zhou [24] found that flavonoid content of soybean seedlings exposed to UV-B treatment during the stress period was enhanced in the beginning and then decreased in comparison with that of the control. Membrane permeability and MDA contents increased at first (first to fifth day) and then decreased (6th–11th day). The main causal factor of the stress damage is free radicals. Flavonoids proved to have the ability to scavenge free radicals.

The excess ROS can react with nearly all cell constituents. Such interaction triggers free radical chain reactions, eventually causing membrane lipid peroxidation. The product of lipid peroxidation, MDA, can react with the amino acid residues of membrane protein and nucleic acid, decline membrane stability, and boost membrane permeability. As a result, cell structure and normal physiological functioning are damaged and accordingly, cell aging and death of organism as a consequence of pathological changes occur. Flavonoids can scavenge surplus free radicals. Under irradiation stress (e.g., gamma, UV), flavonoids improve the plant’s ability of self-protection by inhibiting formation of cellular membrane lipid peroxidation product-MDA and by retaining membrane permeability [24].

## 3. Experimental

### 3.1. Plant Materials

Both accessions were obtained from the Malaysian Agriculture Research and Development Institute (MARDI). Their characteristics are listed in Table 1.

**Table 1:** Leaf morphological characteristics (shape, margin and colour) of two accessions of *C. asiatica.*

Accession code	MARDI code	Leaf shape	Leaf margin	Leaf color
**CA03**	MP09	Kidney-heart	Dentate	Green with purple pigments
**CA23**	MP05	Kidney	Crenate with dentate base	Light green

Young axillary shoots from nodal segments were excised from *in vitro* plantlets of *Centella asiatica* that were cultured in a Duchefa shoot multiplication medium with made up of 2 mg/L BAP and 0.1 mg/L NAA.

### 3.2. Gamma Irradiation and Radiation Sensitivity Test

Irradiation of *Centella asiatica* was conducted in Faculty of Science and Technology, Universiti Kebangsaan Malaysia using a Gammacell 220 Excel Irradiator (MDS Nordion, Ottawa, ON, Canada). The source of gamma rays was Cobalt 60. 

Approximately 1-cm long axillary shoots of *Centella asiatica* were irradiated after five days of culture at 0, 10, 20, 30, 40, 50, 60, 70, 80, 90, 100, and 120 Gy. The Randomize Complete Block Design was used with five replications. After irradiation, the nodes were transferred to fresh Duchefa shoot multiplication medium and were maintained at 25 ± 2 °C and a photoperiod of 16 hours light and eight hours dark. Radiation effect on *Centella asiatica* was recorded in terms of the survival rate (%) after exposure to the gamma radiation. The 50% survival rate was determined. The survival rates (%) of irradiated and non-irradiated plantlets were assessed after three weeks of gamma irradiation.

### 3.3. Induction of Mutation with Selected Doses of Gamma Radiation

The nodal segments were irradiated with gamma rays at does of 30 and 40 Gy for *Centella asiatica* accession ‘CA03’ and 20 and 30 Gy for accession ‘CA23’. The nodal segment response to the radiation was evaluated by recording the flavonoid content, leaf gas exchange and biomass. The experiment was designed as RCBD with five replications.

### 3.4. Plant Harvest and Biomass

Growth measurement and plant harvest were conducted once every two weeks. At each harvest, the leaf biomass per plant was determined after oven-drying the leaves at 45–50 °C to constant mass. 

### 3.5. Determination of the Total Flavonoid Content

The flavonoid contents were determined spectrophotometrically following the method established by Lamaison and Carnat [25] which is based on the formation of a flavonoid-aluminium colored characterized by a wavelength of maximum absorption of 430 nm.

After oven drying of leaf samples at 45 °C–50 °C, they were kept in a −20 °C freezer. Each sample (1 g) was added to 80% methanol (20 mL) and was incubated in Orbit Shaker at 250 rmp and 50 °C for 2 hours. The samples were then filtered. Extract (1 mg) and 2% AlCl_3_ methanol solution (1 mL, 2 mg AlCl_3_ in 100 absolute methanol) was prepared and kept at room temperature for 15 minutes. Finally, the absorbance was measured at 430 nm using spectrophotometer. Rutin was used to create the calibration curve and the flavonoids content were expressed in mg per g of rutin equivalent (mg/g).

### 3.6. Gas Exchange Determination

In the mature and high flavonoid content leaves (eight weeks after planting), the net photosynthetic rate (PN), stomatal conductance (*g*s), intercellular CO_2_ concentration (C_i_), and transpiration rate (E) were determined in the greenhouse using a portable infrared photosynthesis system LI-6400 (LI-COR, Lincoln, USA).

### 3.7. Estimation of Lipid Oxidation (Malondialdehyde (MDA))

The first method of assessing the TBA-MDA complex in plant tissue was offered by Heath and Packer [26]:

MDA equivalents (nmol/mL) = [(A_532_−A_600_)/155,000] × 10^6^

where 532 nm represents the wavelength of maximum absorption of the TBA-MDA complex, A_600_ is a correction for non-specific turbidity, and 155,000 is the molar extinction coefficient for MDA.

## 4. Conclusions

*Centella asiatica* shows a promising prospective for novel discoveries in the commercial and pharmaceutical industry. *In vitro* mutagenesis through gamma radiation can be employed to create economically superior mutants. Gamma irradiations are often applied on plants for developing varieties which are agriculturally and economically important and comprise high productivity and efficiency potential [27]. 

*Centella asiatica* has been recognized to have health benefits and to possess a medicinal value. One of its therapeutic properties is the possession of flavonoids which have antioxidative properties. However, its total flavonoid content is generally inadequate. Some of the significant flavonoids which afford several positive health benefits may be present in very small quantities. For that reason, the potential of enhancing the flavonoids content of *Centella asiatica* was investigated. This study mainly focused on physiological traits and the amounts of total flavonoids in two accessions of *Centella asiatica* at several harvesting times and under different gamma irradiation dosages.

The results indicated that based on the survival percentage, the LD_50_ of CA03 and CA23 plantlets were attendant to 60 and 40 Gy irradiation levels, respectively. Biochemical tests revealed that the irradiated accessions displayed higher total flavonoid content than the non-irradiated ones, though the differences in the mean values among the irradiated accessions were not significant. Moreover, the irradiated plants were found to have the highest flavonoid contents in their eighth week of growth.

Leaf biomass, total flavonoid and leaf gas exchange of two accessions of *Centella asiatica* induced by γ-radiation were compared with control counterparts. Plant leaf biomass was significantly (p < 0.01) inhibited. Decline in net photosynthetic rate, was the result of both stomatal and non-stomatal limitations. Gamma irradiation induces various physiological and biochemical alterations in plants. The irradiation of plants with high dosages of gamma rays causes disorders in the hormone balance, leaf gas exchange, water exchange and enzyme activity [28]. Photosynthetic pigments can be damaged through gamma irradiation with concomitant loss of the photosynthetic ability [29]. It is noteworthy in this study that under gamma conditions the MDA and flavonoid contents increased.

In the future prospects, the *in vitro* propagation of *Centella asiatica* as well as the use of gamma irradiation as elicitor could enable the mass extraction of economically valuable secondary metabolites such as flavonoids which possesses important medicinal properties and are extensively used as a food supplements in many countries. Further investigation on the combined effects of gamma irradiation and CO_2_ enrichment (to compensate detrimental effects of gamma on photosynthetic apparatus) on *Centella asiatica* could be conducted to facilitate the creation of a mutant with superior physiological, agronomical and biochemical qualities for commercial use.
